# A Structural Equation Model for Sustainable Supply Chain Management in the Food Industry †

**DOI:** 10.3390/foods13233713

**Published:** 2024-11-21

**Authors:** Theofilos D. Mastos, Katerina Gotzamani, Petros Ieromonachou, George Tsiotras

**Affiliations:** 1 Business Excellence Lab, School of Business Administration, University of Macedonia, 546 36 Thessaloniki, Greece; kgotza@uom.edu.gr (K.G.); tsiotras@uom.edu.gr (G.T.); 2 Connected Cities Research Group, School of Business, Operations and Strategy, University of Greenwich, London SE10 9LS, UK; p.ieromonachou@gre.ac.uk

**Keywords:** food industry, Greece, structural equation model, sustainable supply chain management

## Abstract

This paper presents a model designed to measure and investigate the relationships between critical factors, practices, and performance of sustainable supply chain management (SSCM) in the food industry. A survey of 423 firms in the Greek food industry was conducted to meet these objectives. The data were analyzed using exploratory factor analysis, followed by confirmatory factor analysis and structural equation modeling, to explore the relationships between the model’s constructs. The results indicate that “firm-level critical sustainability factors” and “supply chain critical sustainability factors” significantly enhance “supply chain collaboration” and “supply chain strategic orientation”. Additionally, “supply chain strategic orientation” positively influences “social performance” and “environmental performance”, while “supply chain collaboration” positively affects “economic performance” and “environmental performance”. Furthermore, “social performance” is found to have a significant positive impact on “economic performance”. This study provides empirical evidence that helps managers understand the importance of the relationships among SSCM critical factors, SSCM practices, and SSCM performance, and enables them to allocate resources effectively and design SSCM strategies. Finally, the developed constructs offer a measurement tool useful for both practitioners implementing SSCM in their firms and researchers who wish to apply or test the proposed scales in other projects or use them as benchmarks.

## 1. Introduction

Over the past two decades, sustainable supply chain management (SSCM) has become a central focus in the business world, drawing significant attention from both academicians and practitioners [1,2]. The globalization of operations has dispersed processes worldwide, linking all supply chain members, through the exchange of information, materials, and capital flows [2]. Various stakeholders, including regulatory bodies, NGOs, community organizations, suppliers, customers, and global competitors, have pressured firms to balance environmental, social, and economic issues in their supply chains [3] through the adoption of sustainable supply chain management practices.

Like any other business operation, SSCM aims to achieve clear performance goals [4]. However, the complexity of the supply chains makes this challenging, as individual members may have different and sometimes even conflicting goals and performance measures. These differing measures may not always positively impact the entire chain’s performance, as one firm’s outcomes can be detrimental to others [5]. As a result, the overall performance of the supply chain can only improve if viewed as a whole, beyond the firm level [6].

SSCM is implemented through specific practices such as environmental purchasing, reverse logistics, and sustainable packaging among others. These practices are anticipated to enhance the sustainability performance of supply chains [7]. The development of SSCM practices can be influenced by various critical factors, which may either enable or inhibit them. Different industries address these factors based on their size, culture, location, and supply chain partnerships. Researchers have studied SSCM across various sectors like manufacturing [2,8], the automotive industry [9], oil and gas [10], energy [11], and the food industry [12]. It is widely accepted, that the food industry faces significant environmental, economic, social, and political challenges such as deforestation, climate change, energy consumption, food safety, production practices, fair wages, and population growth coming to the forefront [12,13].

Other critical SSCM issues include measuring supply chain impacts, collaboration and networking, stakeholder engagement, and sustainable development goals [14]. These challenges highlight the unique variability and risk factors in food supply chains due to product-specific characteristics like perishability, seasonality, and storage conditions [15]. Both customers and firms have expressed concerns about product origins, food safety, product quality, sustainable production [16], animal welfare, and environmental protection [15].

Numerous studies have examined the relationship between SSCM practices and performance to better understand these challenges. In various industries, direct and indirect impacts between sustainability performance dimensions have also been observed. For instance, a positive correlation exists between corporate social performance and financial performance [17]. In the wine industry, ref. [18] found that socially sustainable employee practices reduce costs. The authors of [19] demonstrated that environmental practices lead to positive environmental outcomes and indirectly improve cost performance through quality enhancements. Despite substantial theoretical developments in SSCM, there is still a lack of empirical research on industry and location-specific SSCM critical factors, practices, and performance [12,20,21,22,23]. The food industry, which is characterized by strong supply chain relationships, aims to achieve a high sustainability performance [12].

Based on the above introduction, the following two research gaps have been identified:

- Different industries encounter a single critical factor from varying perspectives, influenced by their size, culture, location, and the number of supply chain members. Research on investigating industry and location-specific SSCM critical factors is still limited [20,21].
- There is also a scarcity of empirical studies on industry-specific SSCM practices and their relationships with SSCM performance.

In this context, this paper aims to address these two gaps by examining the relationships among SSCM critical factors, SSCM practices, and SSCM performance in the Greek food industry.

The rest of the paper is organized as follows: The next section provides an overview of the literature related to SSCM critical factors, SSCM practices, and SSCM performance measures and develops the relative research hypotheses; Section 3 describes the survey methodology that has been utilized; Section 4 presents the results in conjunction with previous research works and discusses the proposed structural equation model. Section 5 concludes the paper and provides the implications and limitations of this study, as well as future research opportunities.

## 2. Literature Review and Research Hypotheses

Since the identification and conceptualization of SSCM is still unclear, a literature review was conducted on three important sustainable supply chain management constructs i.e., SSCM critical factors, SSCM practices, and SSCM performance. The search for relevant scientific articles was conducted using keywords and author names in major bibliographic databases and publishers, such as Scopus, Elsevier, Emerald, Springer, Wiley, Taylor & Francis, Sage Publications, and Inderscience, covering a twenty-year span from 2000 onward. Keywords included terms like “sustainable supply chain management”, “drivers”, “barriers”, “enablers”, “motivators”, “critical factors”, “sustainable supply chain management practices”, “sustainability performance”, and “food industry”. The author search focused on researchers such as Seuring S., Beske P., Gualandris J., Govindan K., and Pagell M., who have consistently contributed to SSCM research [1]. A secondary search was also conducted through cited references. Only peer-reviewed articles in English-language scientific journals were considered. This review primarily focused on studies with applications in the food industry but also included articles from other sectors. The measures identified by the comprehensive literature review are named and grouped based on the affinity method, which is utilized to organize into categories common themes from a large amount of information [24].

### 2.1. SSCM Critical Factors

The critical factors for SSCM are reviewed and categorized into firm-level, supply chain-level, and external critical factors. Firm-level critical factors are defined as organizational factors, while supply chain-level factors encompass factors that are related to the firm’s supply chain environment. External critical factors may originate from a plethora of stakeholders, such as government, customers, suppliers, media, non-governmental organizations (NGOs), etc. These SSCM critical factors are found to be linked with the adoption of SSCM practices.

Previous scholarly works have recognized factors such as top management commitment and proficiency (knowledge and expertise) in sustainability, as significant firm-level determinants in the realm of SSCM. For instance, the absence of commitment and support from top management obstructs the advancement of SSCM [1,5,25]. SSCM requires proactive top management that understands that sustainability is an organizational commitment [26]. Fulfilling customer demands, expectations, and requirements is frequently mentioned as a crucial factor for the successful implementation of SSCM [5,27,28]. Ref. [28] recognized that knowledge and expertise in sustainability are key drivers in developing a firm’s SSCM strategy. Similarly, ref. [27] emphasized that knowledge is a vital intangible asset for implementing SSCM. Other factors that have been highlighted as critical for SSCM in the food industry include operational cost reduction and market, retailer pressure, brand image, and corporate reputation [29].

Supply chain-level critical factors are closely tied to firm-level critical factors. According to the literature, the alignment between firm-level and supply chain-level factors significantly influences their successful integration [30]. Information sharing has been identified as a crucial enabler for adopting SSCM practices [31,32,33]. Ref. [20] suggests that information sharing facilitates the development of new sustainability ideas and enhances collaboration throughout the supply chain. In the food industry, information sharing among supply chain members is seen as a novel method for traceability and is associated with improved supply chain performance [26]. The existing body of literature suggests that the act of sharing information fosters the creation of novel sustainable concepts and amplifies cooperation across the supply chain, as pointed out by [20]. In contrast, the lack of information sharing has a negative impact on SSCM implementation [32,34]. Relationships that are built on trust and commitment among supply chain members are reported as another key factor for SSCM adoption. Ref. [29] explored the implications of sustainability in the Italian meat supply chain and found that trustful relationships are a critical element for deploying exceptional supply chain practices, such as collaboration for sustainability.

Regarding the external environment of critical factors, the identification, engagement and communication with customers, legislative pressures, local community, and NGOs are highlighted as essential elements for effectively executing sustainable supply chain management (SSCM) practices [35]. This corresponds with earlier research, affirming that stakeholders play a pivotal role in propelling the incorporation of SSCM practices [21]. Especially in the food retail industry, NGO pressure is found to be critical for the adoption of SSCM [36].

### 2.2. SSCM Practices

Considering the practices that are adopted in the food industry in order to implement SSCM, five categories are proposed in the literature. The SSCM practices, as proposed by Beske et al. [12] ((1) strategic orientation, (2) supply chain continuity, (3) collaboration, (4) risk management, and (5) pro-activity), are used as a key starting point and as a guiding tool for developing a model of SSCM in the food industry since they cover the aspects of SSCM and focus on strengthening the relationships among supply chain partners, the flow of goods and information, and the sustainability aspects. Strategic orientation is encompassing the commitment of firms to supply chain management and to the triple bottom line (TBL) [12]. Continuity of supply chains involves the development (design and structure) of a supply chain network [12]. Supply chain collaboration, which represents the third SSCM practice, goes beyond the traditional modus operandi between organizations since it is not restricted only to new product development but also to the development and enhancement of business processes [12,37]. Supply chain risk management includes the adoption of risk mitigation practices to avoid exposure to risks, while proactivity refers to the actions taken by a firm in order to control and manage a specific situation regarding sustainability before it happens, rather than responding to it after it happens.

Based on the above, the following research hypotheses have been developed:

**Hypothesis** **1** **(H1).** *SSCM critical factors have a significant positive impact on SSCM practices.*

**Hypothesis** **1a** **(H1a).** *Firm level critical factors have a significant positive impact on SSCM practices.*

**Hypothesis** **1b** **(H1b).** *Supply chain level critical factors have a significant positive impact on SSCM practices.*

**Hypothesis** **1c** **(H1c).** *External critical factors have a significant positive impact on SSCM practices.*

### 2.3. SSCM Performance

SSCM performance refers to how well supply chains achieve their environmental, economic, and social goals. Most studies in the literature focus on the economic and environmental performance aspects, whereas the social dimension and the integration of the three sustainability dimensions are still lagging [2]. Regarding environmental performance, the most frequently used measure is related to either the reduction or avoidance of hazardous/harmful/toxic materials. The second most cited measure is water consumption, followed by energy consumption, recycled materials, life cycle analysis (LCA), and environmental penalties. Energy efficiency, air emissions, and greenhouse gas emissions are reported as some of the most important measures in the literature. The most common measure for assessing economic performance is quality. This can relate to the quality of the purchased products [38] or the quality of the production process itself [39]. Sales, market share, and profit are the next most commonly used metrics, with delivery time and customer satisfaction following. Research indicates that only a few metrics are consistently used, underscoring the limited focus on the social aspect of sustainable supply chain management (SSCM). Among the social metrics, recordable accidents are the most frequent metric, followed by training, education, and labor practices. Several researchers have found that SSCM practices have the ability to enhance environmental and social performance [7,35]. Evidence from the food industry, provided by [29], demonstrates that traceability practices in the meat supply chain are closely associated with social sustainability and food safety.

Based on the above observations, this study developed the following research hypotheses:

**Hypothesis** **2** **(H2).** *SSCM practices have a significant positive impact on SSCM performance.*

**Hypothesis** **2a** **(H2a).** *SSCM practices have a significant positive impact on social performance.*

**Hypothesis** **2b** **(H2b).** *SSCM practices have a significant positive impact on economic performance.*

**Hypothesis** **2c** **(H2c).** *SSCM practices have a significant positive impact on environmental performance.*

Ref. [35] suggests that environmental performance improvements such as energy efficiency practices, have visible cost reductions in the short term. In the same line, for the Italian meat supply chain, ref. [29] found that SSCM practices, such as the adoption of cleaner technologies, confer a competitive edge by enhancing both economic and environmental or social performance. Ref. [40] also found a positive correlation between corporate social performance and corporate financial performance.

Based on the above arguments, this study developed the following two hypotheses:

**Hypothesis** **3** **(H3).** *Environmental performance has a significant positive impact on economic performance.*

**Hypothesis** **4** **(H4).** *Social performance has a significant positive impact on economic performance.*

**Figure 1:**
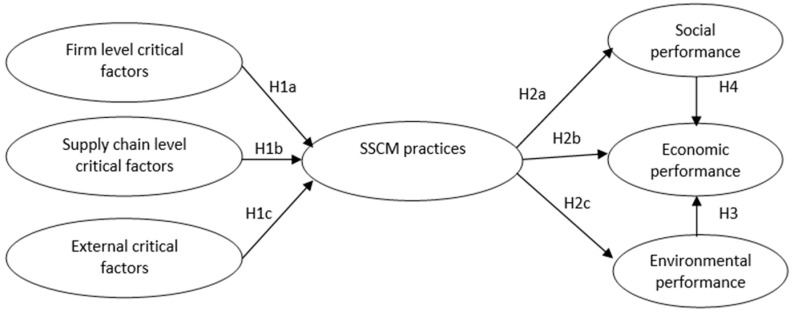
Theoretical model for SSCM in the food industry.

The theoretical developments that have been conducted in the previous paragraphs have assisted in the development of a conceptual model of relations (Figure 1) consisting of three pivotal SSCM concepts: (a) SSCM critical factors; (b) SSCM practices; and (c) SSCM performance. The relations and interactions among these constructs are determined and the research hypotheses are tested in the following sections.

## 3. Research Methodology

The data for this study were gathered through a structured survey questionnaire, which was developed based on a comprehensive literature review on sustainable supply chain management [12,22,27,28,32,33,41,42,43]. Similar research in the SSCM field has also utilized surveys, highlighting the relevance of this approach [44]. The questionnaire was divided into four sections: the first section covered the critical factors for effective SSCM implementation; the second examined various SSCM practices; the third section assessed the SSCM performance; and the fourth focused on firm and respondent profiles. Responses were collected within a period of 8 months and the survey length was approximately 15 min. Content validity was ensured by reviewing the extensive literature, leading to an initial list of 80 items. A pilot study involving 10 food industry experts was then conducted and their feedback was incorporated during the pre-testing phase [45,46] to improve question clarity, define terms, outline the research scope, and refine expected outcomes. The purpose of pilot testing was to examine if each question includes the information that needs to be measured. The detailed expertise support helped capture some industry-specific and cultural issues of the questionnaire. In addition, four academic reviewers also evaluated and revised the draft, resulting in a final list of 68 items [47]. Responses were recorded using a seven-point Likert scale (1 = “strongly disagree” to 7 = “strongly agree”) and data processing was carried out using SPSS 24 and AMOS 21.

### 3.1. Research Sample

The survey sample comprised firms from the food and beverage sector listed in Greek sustainability databases, such as “CSR HELLAS NET”, “Sustainable Greece 2020”, and “CSR Index GR”. Additionally, the sample included firms from other business directories, like the “Federation of Hellenic Food Industries” and ICAP (Greece’s largest business information and consulting firm), resulting in an initial sample of 904 firms. All firms were contacted via email. The questionnaire was aimed at the personnel responsible for supply chain management. It was sent by the authors and university students, who had undergone training relevant to the study’s purpose and content. The email included a cover letter assuring respondents of the confidentiality of their answers and invited them to leave their contact information if they wished to receive the research results. A total of 423 (46.8% response rate) completed questionnaires were received, which was deemed acceptable for data processing, compared to similar studies [22].

### 3.2. Non-Response Bias and Common Method Bias

To evaluate the dataset for non-response bias, the sample was divided into early and late respondents, with the latter group representing theoretical non-respondents, following the approach of ref. [48]. A Mann–Whitney U test was conducted to compare these two groups, and no statistically significant differences were detected, suggesting that non-response bias does not affect this study.

Additionally, common method bias, a significant validity concern in behavioral research [49], was assessed. To mitigate this issue, Harman’s single-factor test was applied to determine if a single factor accounted for more than 50% of the variance in the dataset. The analysis showed that all items loaded in one factor explained only 35.2% of the total variance, hence confirming the absence of common method bias in this study.

## 4. Data Analysis and Results

The sample consisted of firms from various sub-sectors within the food industry, encompassing the entire food supply chain and ensuring the results apply to a broad range of supply chain participants. A total of 40% of the firms operated in multiple supply chain activities, while 22% operated in the food services sub-sector. The rest of the sample included retail firms, food manufacturers, wholesale, beverage producers and, transportation and storage. In terms of firm size more than 87% were small and medium enterprises and 13% were large enterprises with more than 250 employees.

The construct validity of the latent factors was verified through several measures: convergent validity (with AVE > 0.5), discriminant validity (where AVE > Corr^2^ [50,51], face-content validity (obtained via feedback from food industry experts on the questionnaire), and nomological validity (demonstrated by significant correlations among the latent factors identified) [52]. Convergent validity was established by examining the factor loadings (>0.6), the average variance extracted (AVE) (>0.4), and construct reliability (CR) (>0.7) for all constructs [53] (see Table 1). It is worth noting that the AVE value for the construct “Supply Chain Critical Sustainability Factors” was below 0.50. However, when AVE falls between 0.4 and 0.5 and the composite reliability (CR) exceeds 0.6, the construct’s convergent validity is acceptable [54,55]. Discriminant validity was assessed by comparing the AVE with the highest squared correlation between the factor in question and other latent factors [52]. The results showed that the AVE surpasses Corr^2^, which confirms discriminant validity [53].

### 4.1. Modified Research Model and Hypotheses

**Figure 2:**
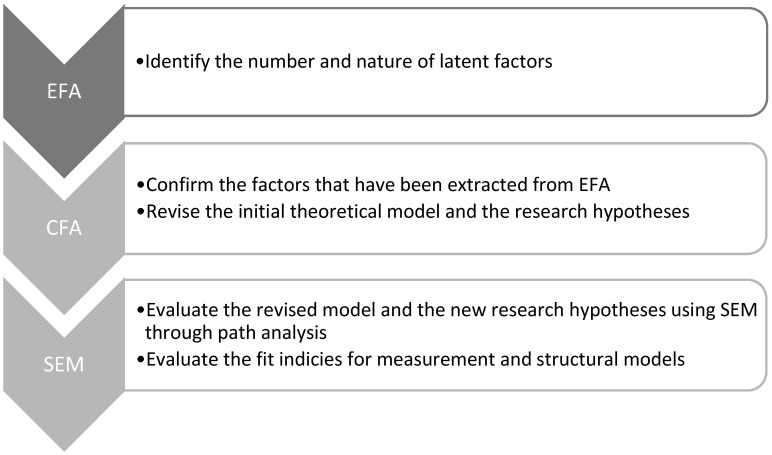
Research stages.

In order to develop a robust structural equation model (SEM), an exploratory factor analysis (EFA) was conducted followed by a confirmatory factor analysis (CFA). The analysis begins with an exploratory factor analysis (EFA), using the principal component extraction method with varimax orthogonal rotation, to identify the structure of the variables. Then, confirmatory factor analysis (CFA) is conducted to refine the scales derived from the EFA and to assess whether the number of factors and the loadings of the measured variables align with the theoretical developments [53]. The necessary research stages, that need to be executed before the application of structural modeling are illustrated in Figure 2.

Based on the EFA, two latent constructs were introduced in the SSCM critical factors category (“firm-level critical sustainability factors” or FLCSF and “supply chain critical sustainability factors” or SCCSF). As a result, H1c from the theoretical model is not supported. Another two factors represented SSCM practices (“supply chain collaboration” or SCC and “supply chain strategic orientation” or SCSO) and three factors reflected the SSCM performance (“environmental” or ENV, “social” or SOC, and “economic” or ECO).

**Figure 3:**
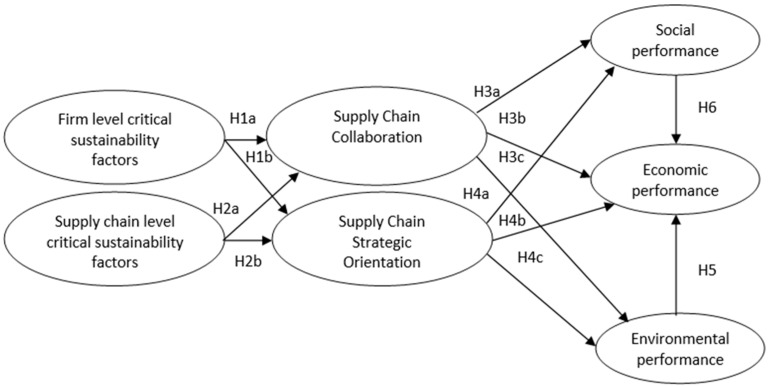
Revised theoretical model for SSCM in the food industry.

In order to investigate the relationships among dependent and independent variables, an SEM technique was applied [53]. Based on the results of the CFA, the study’s theoretical framework has been adjusted to incorporate these changes, now including seven key factors i.e., FLCSF, SCCSF, SCC, SC, ECO, SOC, and ENV. The revised theoretical model is illustrated in Figure 3.

Given the changes to the theoretical model, the original hypotheses regarding the relationships between SSCM critical factors, SSCM practices, and SSCM performance were revised. These modified research hypotheses incorporate the confirmed latent constructs from CFA. Accordingly, new hypotheses were introduced to capture the proposed relationships.

**Hypothesis** **1a** **(H1a).** *Firm level critical sustainability factors have a significant positive impact on supply chain collaboration.*

**Hypothesis** **1b** **(H1b).** *Firm level critical sustainability factors have a significant positive impact on supply chain strategic orientation.*

**Hypothesis** **2a** **(H2a).** *Supply chain level critical sustainability factors have a significant positive impact on supply chain collaboration.*

**Hypothesis** **2b** **(H2b).** *Supply chain level critical sustainability factors have a significant positive impact on supply chain strategic orientation.*

**Hypothesis** **3a** **(H3a).** *Supply chain collaboration has a significant positive impact on social performance.*

**Hypothesis** **3b** **(H3b).** *Supply chain collaboration has a significant positive impact on economic performance.*

**Hypothesis** **3c** **(H3c).** *Supply chain collaboration has a significant positive impact on environmental performance.*

**Hypothesis** **4a** **(H4a).** *Supply chain strategic orientation has a significant positive impact on social performance.*

**Hypothesis** **4b** **(H4b).** *Supply chain strategic orientation has a significant positive impact on economic performance.*

**Hypothesis** **4c** **(H4c).** *Supply chain strategic orientation has a significant positive impact on environmental performance.*

**Hypothesis** **5** **(H5).** *Environmental performance has a significant positive impact on economic performance.*

**Hypothesis** **6** **(H6).** *Social performance has a significant positive impact on economic performance.*

### 4.2. Structural Equation Model

The revised model and the new research hypotheses were evaluated using SEM through path analysis, which is a multivariate analytical approach providing insights into the causal sequence of variables within a network of relationships [56]. According to [57], the measurement and structural models are analyzed sequentially. The process started with a CFA before proceeding to test the hypothesized model [50]. The fit indices for both the measurement and structural models indicated that the data fits well, supporting the theoretical model with empirical evidence [53] (see Table 2). Once the fit of these models was confirmed, the SEM procedures, using the maximum likelihood method, were applied to estimate the causal relationships among latent factors, determining whether to accept or reject the developed hypotheses (H1–H6).

As detailed in Table 3, three hypotheses are fully accepted (H1, H2, and H6), two are partially accepted (H3 and H4) for two out of three unobserved factors, and one (H5) is rejected.

**Figure 4:**
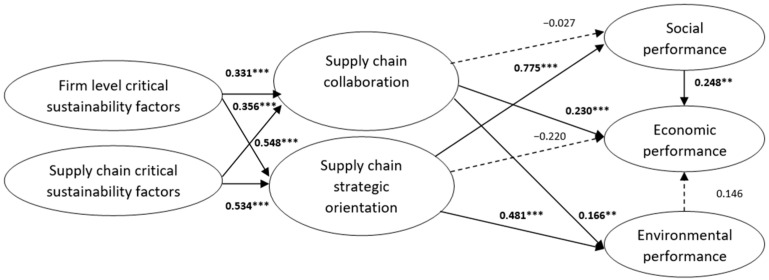
Structural equation model for SSCM in the food industry. Note: ** 0.01 ≤ *p*-value ≤ 0.05; *** *p*-value ≤ 0.01.

The following Figure 4 presents the SSCM structural equation model along with the estimated standardized parameters for the causal paths.

### 4.3. Discussion

The results of this study clearly indicate that critical sustainability factors at both the firm and supply chain levels significantly contribute to supply chain collaboration and strategic orientation. At the firm level, these results align with the work of [58], who emphasize that internal characteristics within the food sector firms can facilitate various supply chain relationships and collaboration opportunities. At the supply chain level, the results are consistent with the studies of [20,29], who highlight that fostering trust among supply chain members and enhancing information sharing can lead to better collaboration. Moreover, critical sustainability factors at both levels positively influence supply chain strategic orientation. For instance, addressing customer demands (as noted by [19,59] and sharing knowledge and information within the supply chain [12] are essential for developing a strategic orientation within the supply chain. Interestingly, external critical factors, such as legislative pressures significantly influence SMEs to integrate sustainable practices into their operations [60], in this study they have been excluded from the revised model. This is probably due to the fact that supply chain critical sustainability factors include items that are related to supply chain partnerships and this might have been perceived by the participants as external driving forces.

In line with previous research [61], this study provides empirical evidence of a positive link between SSCM practices and SSCM performance within the Greek food supply chain sector. The results indicate that SSCM practices, such as supply chain collaboration [62,63] and strategic orientation, significantly contribute to sustainability performance.

This study is based on the SSCM theory as proposed by [64]. The theory suggests that the triggers for SSCM involve stakeholder management and pressures, drivers, and barriers of SSCM. The supplier management for risk and performance element incorporates the multi-tier supplier management, the supplier selection and evaluation and the risk and performance outcomes. The third and last part of SSCM theory is related to the supply chain management for sustainable products, which involves supplier development, communication, and collaboration that target the development of sustainable products.

Consistent with SSCM theory as explored by [2,13,26,64], the results show that fostering collaboration through strengthened supply chain relationships can enhance both economic [61] and environmental performance in the food industry. However, the study found a non-significant negative relationship between supply chain collaboration and social performance, which contrasts with the findings of [22], who observed a significant link between SSCM practices and social performance. This discrepancy may stem from the fact that, while collaboration may bring economic and environmental benefits in the short term, social sustainability improvements typically require a longer timeframe to materialize.

Consistent with the findings of [22], who explored the connections between SSCM practices and SSCM performance, this study found a positive relationship between supply chain strategic orientation and both environmental and social performance. However, the results differ from [22] regarding the economic performance, where a non-significant negative relationship is observed. This suggests that the economic impacts of the strategic orientation may not be immediately apparent. Additionally, it indicates that employees and managers in the food industry recognize the environmental and social benefits but may be less aware of, or even skeptical about, potential positive economic outcomes. This result implies that when food supply chain firms adopt more sustainability-focused practices, there may be higher initial costs, which could negatively affect economic performance in the short term. This is also confirmed by [62], who found that the cost of sustainability may hinder the adoption of SSCM practices in multi-tier food supply chains.

The structural equation model also examined the interconnections of the sustainability performance dimensions. The results indicated that social performance has a significant positive influence on economic performance. This aligns with [40], who found a positive correlation between corporate social performance and corporate financial performance. Additionally, this result supports the case study of exemplars presented by [35], which demonstrated that social performance is positively linked to economic performance. The social performance indicators suggest a focus on safety (product, health, and safety), leading to the conclusion that social performance is connected to safety management. This implies that improving safety aspects (i.e., social performance) can enhance economic performance by lowering costs related to safety issues. On the other hand, environmental performance was found to have a positive but statistically insignificant effect on economic performance. This is consistent with [19], who observed that environmental performance does not directly impact costs in the food industry. Although the case study conducted by [35] suggests that improvements in environmental performance can yield economic benefits through short-term cost reductions, the respondents expressed a different opinion. It is worth noting that 87% of the sample consisted of SMEs, which often lack the resources to prioritize costly environmental improvements. Therefore, it is reasonable to conclude that Greek firms in the food supply chain still have considerable progress to make in raising environmental awareness and recognizing the long-term benefits and opportunities that could indirectly enhance economic performance. A potential regional factor that might explain this result is the prolonged Greek economic crises (encompassing the debt crisis, capital controls, and COVID-19), which have compelled companies fighting for survival to reduce or even eliminate costs deemed “unnecessary” or considered as “luxuries”.

In order to facilitate the understanding of the results and provide a visualization for professionals in the SSCM field, a summary of the key results is presented in Table 4.

## 5. Conclusions

This study contributes to the existing sustainable supply chain management research by introducing a measurement tool that encompasses three key SSCM concepts: critical factors, practices, and performance. The first component examines the crucial elements firms need to consider for successful SSCM implementation. The second focuses on factors related to actual SSCM practices, while the third addresses factors necessary for improving SSCM performance. These constructs offer valuable insights specifically within the context of the food industry in Greece. A structural equation model was developed to test the relationships between these three constructs, offering insights that future studies can either validate or challenge.

A key conclusion from this research is that food firms aiming to effectively implement sustainable supply chain management must focus on three crucial areas, which form the foundation of the factors requiring attention. The study demonstrated a positive influence of both firm-level and supply chain-level critical sustainability factors on SSCM practices, such as supply chain strategic orientation and collaboration. It also highlighted the positive impact of effectively implementing SSCM practices on SSCM performance. Additionally, the research revealed a positive link between social and economic performance in food supply chains. The study presents a theoretically developed model, which was empirically tested and proved to be reliable and valid for exploring the relationships among critical factors, practices, and performance in SSCM. This model can serve as a self-assessment or benchmarking tool for firms and supply chain and sustainability professionals in the food industry.

In line with other research studies [65], this paper also contributes to two UN Sustainable Development Goals (SDGs). In terms of Zero Hunger (SDG 2), this study provides a model that helps decision-makers design sustainable practices and food systems from a supply chain perspective. Regarding Climate Action (SDG 13), the developed SSCM model is expected to assist in the reduction of vulnerability and strengthen climate resilience. The link between the results of this study and the UN SDGs, highlights the SSCM’s ability to alleviate environmental, social, and economic pressures, while advancing sustainable development [66].

This research specifically focused on food enterprises in Greece, and, likely different SSCM critical factors, SSCM practices, and SSCM performance metrics may emerge in other industries or regions. Future studies could broaden their scope by including diverse industries and countries, and using larger sample sizes to improve the generalizability of the results. Finally, since this study was conducted during the COVID-19 pandemic, a valuable research opportunity lies in replicating the survey in the post-pandemic era. This could provide insights into how the pandemic may have influenced SSCM practices and performance within the context studied.

## Figures and Tables

**Table 1:** Reliability and convergent validity.

Latent Factors	Items	Factor Loadings	AVE	CR	Cronbach’s Alpha
Firm-level Critical Sustainability Factors	4	0.741	0.549	0.829	0.817
Supply Chain Critical Sustainability Factors	3	0.656	0.431	0.694	0.706
Supply Chain Collaboration	4	0.745	0.561	0.835	0.824
Supply Chain Strategic Orientation	3	0.710	0.504	0.753	0.776
Economic Performance	4	0.880	0.765	0.929	0.932
Social Performance	6	0.730	0.536	0.873	0.877
Environmental Performance	3	0.729	0.535	0.774	0.770

**Table 2:** Acceptable fit indices for the measurement and structural model.

Fit Indices	Measurement Model	Structural Model	Acceptable Fit Indices
Absolute fit indices			
Chi-square (CMIN or χ^2^)	666.624	513.701	0 ≤ χ^2^ ≤ 2df
Degrees of freedom (df)	301.000	216.000	
Probability level	0.000 *	0.000 *	*p* > 0.05
Root mean square residual (RMR)	0.075	0.071	<0.08
Root mean square of approximation (RMSEA)	0.056	0.060	<0.08
Incremental fit indices			
Incremental fit index (IFI)	0.937	0.937	>0.90
Tucker–Lewis coefficient (TLI)	0.925	0.926	>0.90
Comparative fit index (CFI)	0.936	0.937	>0.90
Parsimonious fit indices			
Chi-square/degrees of freedom (χ^2^/df)	2.215	2.378	Between 1 and 3
Normed fit index (NFI)	0.890	0.897	>0.50
Goodness of fit index (GFI)	0.889	0.901	>0.50
Adjusted goodness of fit index (AGFI)	0.861	0.874	>0.50

Note: * Acceptable when *n* > 250, the number of the measured variables range between 12 and 30, RMR < 0.08, RMSEA < 0.07, and CFI > 0.92 [48].

**Table 3:** Relationships between constructs.

Relationships	Standardized Regression Weights	Standard Error	*p*-Value	Hypothesis Test Result
H1a: FLCSF -> SCC	0.331	0.127	0.000	Accept hypothesis
H1b: FLCSF -> SCSO	0.548	0.083	0.000	Accept hypothesis
H2a: SCCSF -> SCC	0.356	0.177	0.000	Accept hypothesis
H2b: SCCSF -> SCSO	0.534	0.118	0.000	Accept hypothesis
H3a: SCC -> SOC	−0.027	0.043	0.685	Reject hypothesis
H3b: SCC -> ECO	0.230	0.089	0.003	Accept hypothesis
H3c: SCC -> ENV	0.166	0.077	0.029	Accept hypothesis
H4a: SCSO -> SOC	0.775	0.074	0.000	Accept hypothesis
H4b: SCSO -> ECO	−0.220	0.189	0.080	Reject hypothesis
H4c: SCSO -> ENV	0.481	0.108	0.000	Accept hypothesis
H5: ENV -> ECO	0.146	0.086	0.056	Reject hypothesis
H6: SOC -> ECO	0.248	0.178	0.015	Accept hypothesis

**Table 4:** Key results.

Key Result Area	Description
SSCM critical factors	Internal characteristics within the food sector firms facilitate supply chain relationships and collaboration opportunities
	Trust and enhanced information sharing among supply chain members lead to better collaboration
SSCM practices and performance	Addressing customer demands and sharing knowledge and information within the supply chain are essential for developing a strategic orientation
	Fostering collaboration through strengthened supply chain relationships enhances economic and environmental performance
	Social sustainability improvements due to improved collaboration typically require a longer timeframe to materialize
	Supply chain strategic orientation positively influences environmental and social performance
SSCM performance	Social performance has a significant positive influence on economic performance
	Greek firms in the food supply chain still have considerable progress to make in raising environmental awareness and recognizing the long-term benefits and opportunities that could indirectly enhance economic performance.

## Data Availability

The original contributions presented in the study are included in the article, further inquiries can be directed to the corresponding author.
